# Immune Effect of T Lymphocytes Infiltrated by Tumors on Non-Small-Cell Lung Cancer

**DOI:** 10.1155/2022/4662874

**Published:** 2022-09-10

**Authors:** Siyuan Sheng, Chuangang Lu, Jianhui Guo, Minjing Liu, Yongdong Wu

**Affiliations:** ^1^Department of Basic Medicine, Xiangnan University, Chenzhou 423000, Hunan, China; ^2^Sanya Central Hospital, Sanya 572000, Hainan, China; ^3^Spine Surgery of Changde Second People's Hospital, Changde 415000, Hunan, China

## Abstract

Lung cancer is increasing every year and it has high morbidity and mortality. Antitumor immunotherapy is a new method for the treatment of lung cancer. Currently, tumor immunotherapy mainly includes classical immunotherapy and immune-targeted therapy To explore the influence of tumor T-lymphocyte (T-cell) infiltration in non-small-cell lung cancer (NSCLC) patients, 100 NSCLC patients diagnosed and treated in Changde Second People's hospital were recruited. Patients were followed up for 3 years. The subjects were divided into a survival group (group S) and a death group (group D). The patient's pathological tissue sections were made, and the degree of T-cell infiltration was counted by H&E (Hematoxylin and eosin) staining. The infiltration degree was graded, and the positive rate of T-cell subsets was calculated by immunohistochemical staining. The 3-year positive rate was 48%, with 48 cases in group S and 52 cases in group D. The positive rate of H&E staining of group S was 100%, including 0 cases of grade 0, 5 cases of grade 1 (10.42%), 16 cases of grade 2 (33.33%), and 27 cases of grade 3 (56.25%). The positive rate of group D was 86.54%, including 4 cases of grade 0 (8.89%), 10 cases of grade 1 (22.22%), 25 cases of grade 2 (55.56%), and 6 cases of grade 3 (13.33%). The total number of T-cell infiltrates in group S was much higher than that in group D (*P* < 0.05). Immunohistochemical results showed that the mean positive rate of CD8^+^ T-cell infiltration was 72.1% in group S and 47.6% in group D, with a considerable difference (*P* < 0.05). No remarkable difference was found in CD4^+^ and CD25^+^ (*P* < 0.05). CD8^+^ + CD4^+^, CD8^+^/CD4^+^, CD25^+^/CD8^+^, CD25^+^/CD4^+^, and CD25^+^/(CD8^+^ + CD4^+^) positive rates were calculated, and the difference between group S and group D was substantial in CD8^+^ + CD4^+^ (*P* < 0.05). The results showed that T cells infiltrated by tumors had an immunosuppressive effect on tumor cells.

## 1. Introduction

Lung cancer is a malignant tumor with high morbidity and mortality that occurs in the upper bronchial mucosa, and its morbidity and mortality are increasing every year [1–3]. In 2020, the morbidity and mortality of lung cancer patients in China were 37.0% and 39.8%, respectively, worldwide, with 816,000 new cases, accounting for 17.9% of new cancers in China [4]. The number of deaths were 715,000, accounting for 23.8% of the total deaths due to malignant tumors in China. Among them, 80%–85% of lung cancer patients have non-small-cell lung cancer (NSCLC). NSCLC includes adenocarcinoma, squamous cell carcinoma, and large cell carcinoma, and treatment is generally carried out according to clinical stage [5–7]. The survival time of lung cancer patients is closely related to clinical stage. Early screening and diagnosis can improve the positive rate of NSCLC patients and reduce mortality. Since 2005, China has carried out malignant tumor screening programs, including lung cancer, in rural and urban areas. It aims to improve the medical level and strengthen the publicity of tumor knowledge (including the symptoms of different tumors, examination methods, and prevention measures), improve the national lung cancer screening rate, and achieve early detection and early treatment. Despite the continuous progress of medical technology and the increasing number of treatment methods in recent years, the positive rate of NSCLC patients is still not high. Some studies have found that the 5-year positive rate is less than 20%, and local recurrence and distant metastasis are the two main reasons leading to the high mortality of patients [8, 9].

At present, the main treatments include surgery, radiotherapy, chemotherapy, and immunotherapy. The tumor immune microenvironment is closely related to the occurrence and development of tumors. Immune cells, especially the T lymphocyte subpopulation, maintain normal immune function and monitor the body's immune function. Immune suppression lead to the tumors formation. The factors that affect mutant cells to avoid immune surveillance mainly include tumor immunosuppression and tolerance. The first kind is tumor immunosuppression. Tumors induce the body to produce immunosuppressive cells and factors, such as regulatory T cells (Treg) and myeloid-derived suppressor cells (MDSCs). Tregs are produced by CD4^+^ and CD25^+^ T-cell subsets in the thymus to maintain the immune function and stability of the internal environment. These cells account for 2%–5% of peripheral T cells. Tregs inhibit T-cell function and secrete immunosuppressive factors to promote tumors. Bone marrow cells are derived from MDSCs, a population of cells that can accumulate gradually in cancer patients and suppress immune function. The second is tumor immune tolerance. Due to the lack of one or more components to stimulate the body's immune system, tumor cells have low immunogenicity and even induce the death of immune functional cells [10–14].

Antitumor immunotherapy is a new method for the treatment of lung cancer. Currently, tumor immunotherapy mainly includes classical immunotherapy and immune targeted therapy [15–20]. Classical immunotherapy generally includes three strategies, namely, active, passive, and supportive immunotherapy. The first is active immunotherapy. By injecting a tumor vaccine, CD4^+^ and CD8^+^ effector T cells can be induced to clear tumor cells in the patient and enable host cells to avoid attack. At present, many tumor vaccines have been developed in the clinic, including antigen specific, tumor cell, and DC cell vaccines [21]. The second is passive immunotherapy. The immune effector is produced outside the body and delivered into the tumor patient. The most common forms are injections of recombinant cytokines, immune effector cells, and monoclonal antibodies [22]. The efficacy of immunotherapy generally does not decrease with advanced age, but the efficacy generally decreases in patients with autoimmune diseases and long-term use of hormone-based drugs [23].

The parameter, which is used commonly for evaluating anti-tumor efficacy, is the Response Evaluation Criteria in Solid Tumors (RECIST) for solid tumors [24]. Patients were graded into complete response (CR), partial response (PR), progressive disease (PD), and stable disease (SD) rates. However, it is now evaluated that this standard evaluation is not a good evaluation of the efficacy of immunotherapy for tumor treatment. For example, RECIST can be evaluated as PD after immunotherapy, which may change to CR, PR, or SD. Therefore, researchers have proposed a new efficacy evaluation criterion, the immune-related Response Criteria (IRRC), which can more accurately and objectively evaluate the efficacy of tumor immunotherapy [25]. Tumor-infiltrating T cells have been proven to be a predictor of the efficacy of immunotherapy by many studies. Anti-PD-1 mainly inhibits tumor proliferation by inducing tumor infiltration of CD8^+^ T cells, while anti-CTLA-4 plays an immunotherapy role by inducing CD8^+^ effector T cells and CD8^+^ T-cell proliferation. The content of CTLA-4 +PD-1+ tumor-infiltrating cells is correlated with the therapeutic effect of PD-1, and the higher the content is, the stronger the effect [26].

Studies have shown that approximately two-third of the infiltrated cells in the stroma of NSCLC tumors are lymphocytes, and approximately 80% of the lymphocytes are T cells [27]. Tumor-infiltrating lymphocytes (TILs) refer to T cells that mainly exist in the local part of the tumor. Lymphocytes are immersed in the tumor microenvironment to participate in tumor immunity, and TILs can suppress tumor growth to a certain extent, which represents the body's anti-tumor immune response. TILs with different densities have different prognostic effects on tumors. Studies have confirmed that certain types of TILs, such as CD4^+^ T and CD8^+^ T cells, can inhibit tumor growth and have immune effects on tumor patients. It was found that the total number of tumor-infiltrating T cells was related to the prognosis of the tumor due to the immune effect on the tumor, and the tumor-infiltrating T-cell subpopulation also had a strong relationship with the prognosis. Abnormal immune function will weaken the body's ability to defend against tumors and cause abnormal proliferation and diffusion of tumor cells, so the immune function of most patients with malignant tumors will have problems. Studies have confirmed that T-cell dysfunction can occur and fail to inhibit tumor growth, even with a high degree of tumor invasion [28]. Tumor-infiltrating T cells are populations containing many different subsets of cells, which can be divided into different subsets according to different differentiated antigens, including five types, namely, CD3^+^, CD4^+^, CD8^+^, CD16^+^, and CD25^+^. Under normal circumstances, the ratios of TILs and T-cell subsets from different tumor sources are different, but the ratios remain constant and cooperate and restrict each other to maintain the normal immune function of the body. Higher or lower ratios of either of them will lead to immune disorders. For example, CD8^+^ T cells increased and the CD4^+^/CD8^+^ ratio decreased in infiltrating lymphocytes of oral squamous cell carcinoma. The lower the degree of differentiation, the lower the ratio. The content of CD25^+^ T cells in freshly isolated tumor-infiltrating T cells was low, and the percentage of CD25^+^ T-cells increased with increasing CD25^+^ T cells In Vitro. CD3^+^ subsets represent the immune function of T cells in the body and are mainly composed of CD4^+^ and CD8^+^ cells. CD4^+^ T cells represent helper T cells (Th), which can assist cellular immunity, while CD8^+^ cells represent inhibitory T cells (Ts), which can inhibit humoral immunity. CD8^+^ cells are proposed to be the effector cells of the body's immune cells that directly kill the tumor, and the more CD8^+^ cells there are, the better the prognosis of the tumor. Observing the status of tumor-infiltrating T cells in NSCLC patients can analyze their immune effect on tumors and analyze the development of tumors [29–35].

## 2. Materials and Methods

### 2.1. The Research Objective

One hundred NSCLC patients diagnosed and treated in Changde Second People's hospital from December 2017 to December 2018 were recruited. Patients were followed up for 3 years. The inclusion criteria for patients is as follows: (i) patients aged ≥18 years; (ii) patients diagnosed with NSCLC by pathological examination and imaging examination; and (iii) patients without preoperative chemoradiotherapy. The exclusion criteria for patients is (i) patients with other malignant tumors; (ii) patients with heart, liver, kidney, and other life-threatening diseases; (iii) patients with certain mental diseases who could not cooperate with the experiment on their own; and (iv) patients with incomplete or lost clinical data. All subjects signed informed consent forms, and the study was approved by the ethics committee of Changde Second People's hospital.

### 2.2. Main Instruments and Reagents

Table 1 shows the reagents used for the detection of various CD T cells.

### 2.3. The Experimental Methods

The survival of patients was followed up by telephone or letter. The total follow-up time was 3 years, and the survival of patients over 3 years was recorded. The study subjects were divided into group S and group D, and the positive rate of patients was counted.

Three years later, pathological specimens of the patients were collected, and tissue sections were made. H&E staining was performed to calculate the degree of T-cell infiltration and grade the infiltration degree. Specific indicators are shown in Table 2. The positive rates of CD8^+^, CD4^+^, and CD25^+^ lymphocyte subsets were calculated by immunohistochemical staining.

- Section dewaxing. Paraffin was removed from tissue sections by xylene and placed in ethanol gradient concentration solution (80%, 70%, 50%) and distilled water for 1 min each.
- H&E staining. After section dewaxing process, the staining of nucleus was done by placing section in hematoxylin dye solution for 10 min and then washed with water for 5 min. In the separation, 1.0% hydrochloric acid + ethanol solution was used for 30 s, and then washed with water for 30 s. Blue staining was done by dilute lithium carbonate solution for 1 min. After staining, samples were washed with water. Cytoplasm staining was done with 0.5% eosin solution for 1 min, followed by washing. 50%, 70%, 80%, and 95% gradient concentration ethanol solution was used for gradual dehydration for 1 min, xylene was used to make the section transparent (neutral gum seal). After that microscopic observations were done.
- Immunohistochemical staining. Section dewaxing was performed and sections were incubated with 3% H_2_O_2_ at room temperature for 10 min and then rinsed with distilled water, Sections were immersed with PBS for 5 min (repeated twice). Primary antibody working solution (anti-CD4, CD8, CD25 mAb) was added and incubated at 37°C for 1.5 h. After that, sections were rinsed with PBS for 2 min (repeat rinsing 3 times), secondary antibody working solution was added and incubated at 37°C for 30 min. Rinsing with PBS for 2 min (repeat rinsing 3 times) was done and chromogenic agent for 10 min (DAB solution) added, which was followed by rinsing with tap water, redye, transparent, and sealed. At the end, microscope observation was performed.

(1) $$\begin{array}{c} \mathrm{Accumulate}\;\mathrm{survival}\;\mathrm{rate}=\frac{\mathrm{Number}\;\mathrm{of}\;\mathrm{survivors}}{\mathrm{Total}\;\mathrm{number}}*\mathrm{100\%,}\\ \mathrm{Positive}\;\mathrm{rate}\;\mathrm{of}\;\mathrm{HE}\;\mathrm{staining}=\frac{\mathrm{Number}\;\mathrm{of}\;\mathrm{cases}\;\mathrm{with}\;\mathrm{lymphocyte}\;\mathrm{infiltration}}{\mathrm{Total}\;\mathrm{number}\;\mathrm{of}\;\mathrm{this}\;\mathrm{group}}*\mathrm{100\%,}\\ \mathrm{Immunohistochemical}\;\mathrm{staining}\;\mathrm{positive}\;\mathrm{rate}=\frac{\mathrm{Number}\;\mathrm{of}\;\mathrm{positive}\;\mathrm{cells}\;\mathrm{in}\;\mathrm{1000}\;\mathrm{lymphocytes}/\mathrm{number}\;\mathrm{of}\;\mathrm{random}\;\mathrm{number}}{\mathrm{1000}\;\mathrm{lymphocytes}\;\mathrm{in}\;\mathrm{field}\;\mathrm{of}\;\mathrm{vision}}*\mathrm{100\%}. \end{array}$$

### 2.4. The Counting Methods

H&E staining was used to display the counts of CD8^+^ T cells, CD4^+^ T cells, and CD25^+^ T cells. T-cell-positive cells were stained tan-yellow. Staining numbers of CD8^+^ T cells, CD4^+^ T cells, and CD25^+^ T cells in each field were counted. The three visual fields were selected, and the count was averaged. The colored cells were pressed, the upper line and the right line were counted, and the lower line and the left line were discarded.

The method used to determine the immunohistochemical results is as follows. Pathological sections were observed under a high-power microscope (×400). The field with more tumor cells and fewer tumor stroma and normal cells was selected, and the field with more tumor stroma and fewer tumor cells was selected. Three fields were randomly selected, each field was counted three times, and the average value was taken.

**Figure 1:**
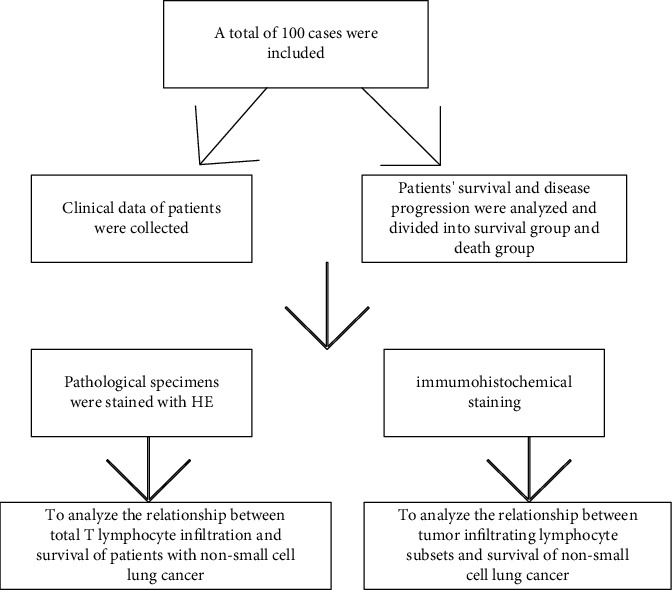
Technical flowchart to analyze the relationship between T-cell infiltration and survival of S and D group.

The technical route followed in this study is shown in Figure 1.

### 2.5. Statistical Methods

All the data were analyzed by SPSS 26.0. The counting data were indicated with a percentage (%), tested using the *χ*^2^(Chi-squared) test. *P* < 0.05 was considered statistically significant.

## 3. Results

### 3.1. Statistical Results of Patient Survival

**Figure 2:**
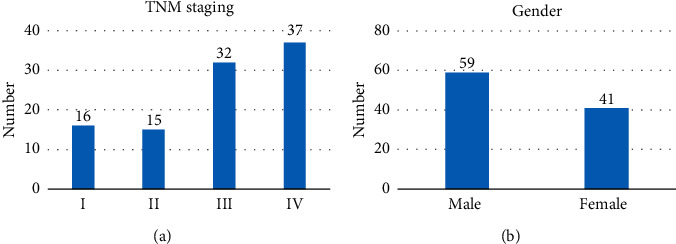
Patient general data statistical results. (a) TNM staging of patients; (b) the sex distribution of patients.

**Figure 3:**
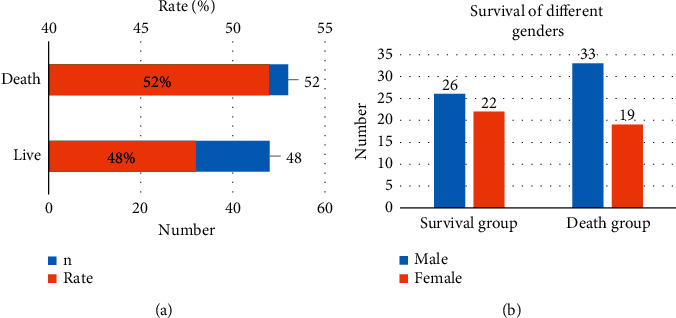
Statistical results of the 3-year survival of patients. Note: (a) represents the overall survival situation; (b) represents the survival of patients of both sexes.

There were 100 patients with TNM stages as follows. There were 16 patients in stage I, 15 in stage II, 32 in stage III, and 37 in stage IV. There were 59 male patients and 41 female patients. The average age was 59.2 ± 4.51 years (Figure 2). Forty-eight patients (26 males and 22 females) survived, and 52 patients (33 males and 19 females) died at 3 years, with an SR of 48% (48/100). The 100 patients were divided into group S and group D according to 3-year survival (Figure 3).

### 3.2. Relationship between Total T-Cell Infiltration and the Postoperative Survival Rate of NSCLC Patients

**Figure 4:**
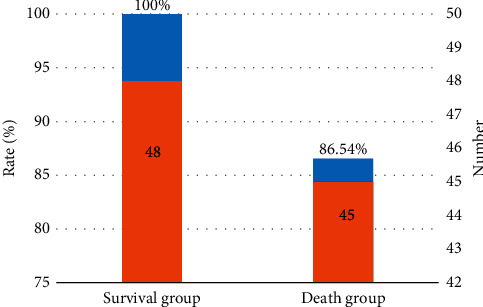
Comparison of the total number and positive rate of infiltrating lymphocytes between group S and group D.

**Figure 5:**
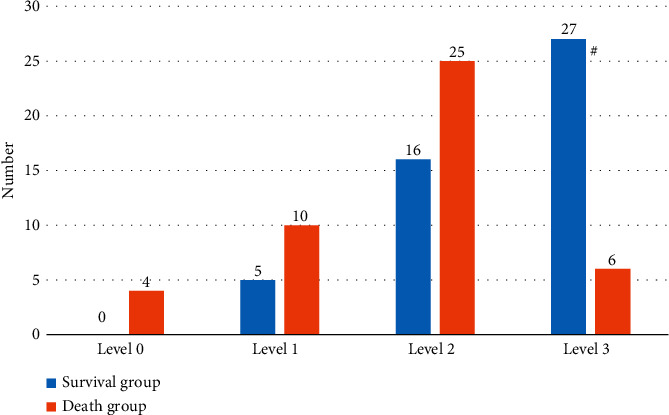
Comparison of lymphocyte infiltration grade between group S and group D. Note: ^#^indicates that the total number of T-cell infiltrates in group S was considerably higher than that in group D, *P* < 0.05.

Patients in group S (48 cases) had lymphocyte infiltration in pathological tissue sections, with a positive rate of 100%, including 0 cases of grade 0, 5 cases of grade 1 (10.42%), 16 cases of grade 2 (33.33%), and 27 cases of grade 3 (56.25%). In patients in group D (52 cases), 7 cases did not have lymphocytic infiltration, and 45 cases had lymphocytic infiltration, with a positive rate of 86.54%, including 4 cases of grade 0 (8.89%), 10 cases of grade 1 (22.22%), 25 cases of grade 2 (55.56%), and 6 cases of grade 3 (13.33%). The total number of T-cell infiltrates in group S was notably higher than that in group D (*P* < 0.05) (Figures 4 and 5).

### 3.3. Relationship between CD8^+^, CD4^+^, CD25^+^ T-Cell Infiltration and the Postoperative Survival Rate of NSCLC Patients

**Figure 6:**
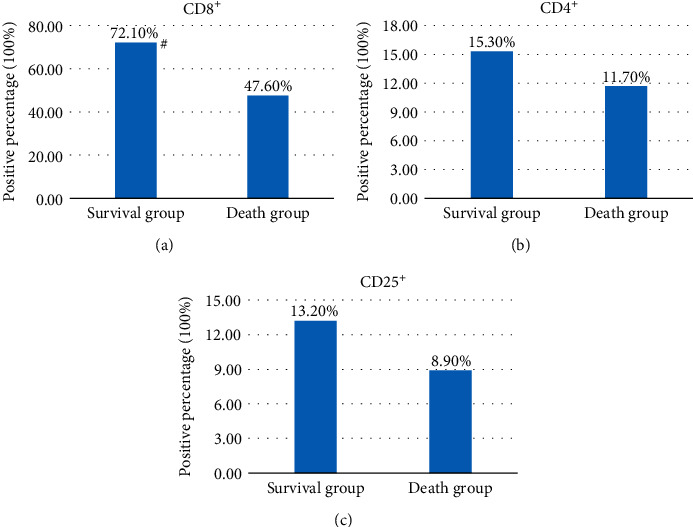
Relationship between CD8^+^, CD4^+^, and CD25^+^ cell infiltration and postoperative positive rate in NSCLC patients. (a) The relationship between CD8^+^ cell infiltration and postoperative positive rate of NSCLC patients; (b) the relationship between CD4^+^ cell infiltration and postoperative positive rate of NSCLC patients; (c) the relationship between CD25^+^ cell infiltration and postoperative positive rate NSCLC patients. ^#^indicates that the average positive rate of the CD8^+^ subgroup in group S was substantially higher than that in group D (*P* < 0.05).

Immunohistochemistry was performed on pathological sections of all patients with CD8^+^ monoclonal antibody, and the positive cells were brown-yellow. In Figure 6(a), the average positive rate of patients in group S (48 cases) was 72.1% and that of patients in group D (52 cases) was 47.6%. The average positive rate of the CD8^+^ subgroup in group S was obviously superior to that in group D (*P* < 0.05). Immunohistochemistry was performed on pathological sections of all patients with CD4 mAbs, and the positive cells were brown-yellow. The results showed that the average positive rate of group S (48 cases) was 15.3%, and the average positive rate of group D (52 cases) was 11.7%. The average positive rate of CD4^+^ subsets in group S was higher than that in group D, and the difference was not remarkable (*P* > 0.05) (Figure 6(b)). Immunohistochemistry was performed on pathological sections of all patients with CD25 mAbs, and the positive cells were brownish yellow. The results in Figure 6(c) show that the average positive rate of group S (48 cases) was 13.2%, and the average positive rate of group D (52 cases) was 8.9%. The average positive rate of the CD25^+^ subgroup in group S was superior to that in group D, but the difference was not substantial (*P* > 0.05).

### 3.4. Relationship between CD8^+^ + CD4^+^ and CD8^+^/CD4^+^ and the Postoperative Survival Rate of NSCLC Patients

**Figure 7:**
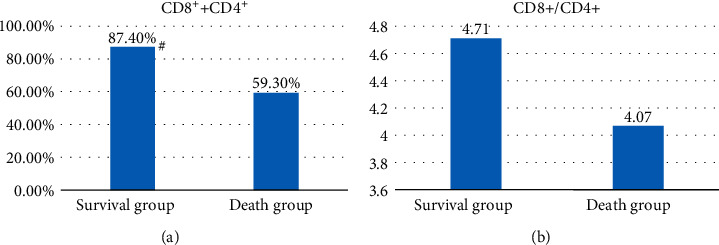
Relationship between CD8^+^ +CD4^+^, CD8^+^/CD4^+^ and postoperative positive rate in NSCLC patients. (a) The sum of the positive percentages of CD8^+^ T cells and CD4^+^ T cells; (b) the ratio of CD8^+^ T cells to CD4^+^ T cells. ^#^indicates that the degree of T-cell infiltration in group S was higher than that in group D, and the difference was statistically significant (*P* < 0.05).

The sum of CD8^+^ T cells and the positive percentage of CD4^+^ T cells (CD8^+^ + CD4^+^) represents the degree of T-cell infiltration.  Figure 7(a) shows that the average degree of T-cell infiltration in group S (48 cases) was 87.4% and that in group D (52 cases) was 59.3%. The degree of T-cell infiltration in group S was evidently higher than that in group D (*P* < 0.05).  Figure 7(b) shows that the ratio of the CD8^+^/CD4^+^ T-cell positive percentage in group S (48 cases) was 4.71 on average and that in group D (52 cases) was 4.07 on average. Group S was higher than group D (*P* < 0.05).

### 3.5. Relationship between (CD25^+^/CD8^+^) (CD25^+^/CD4^+^) (CD25^+^/(CD8^+^ + CD4^+^)) and Postoperative Survival Rate of NSCLC Patients

**Figure 8:**
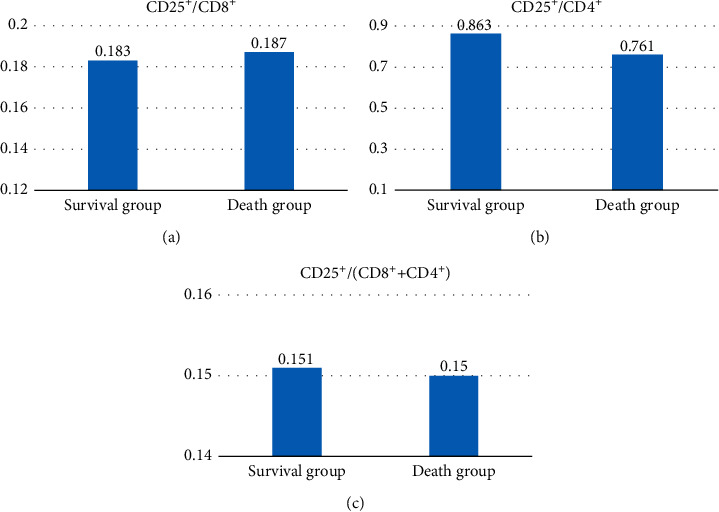
Relationship between (CD25^+^/CD8^+^), (CD25^+^/CD4^+^), (CD25^+^/(CD8^+^ + CD4^+^) and postoperative positive rate in NSCLC patients. (a) The relationship between CD25^+^/CD8^+^ and the postoperative positive rate of NSCLC patients; (b) the relationship between CD25^+/^CD4^+^ and the postoperative positive rate of NSCLC patients; (c) the relationship between CD25^+^/(CD8^+^ + CD4^+^) and the postoperative positive rate of NSCLC patients.

In Figure 8(a), the average CD25^+^/CD8^+^ T-cell ratio of group S (48 cases) was 0.183, and the average CD25^+^/CD8^+^ T-cell ratio of group D (52 cases) was 0.187, showing no significant difference (*P* > 0.05). In Figure 8(b), the average CD25^+^/CD4^+^ T-cell ratio of group S (48 cases) was 0.863, and the average CD25^+^/CD4^+^T-cell ratio of group D (52 cases) was 0.761, showing no significance (*P* > 0.05).  Figure 8(c) shows that the average CD25^+^/(CD8^+^ + CD4^+^) T-cell ratio of group S (48 cases) was 0.151, and the average CD25^+^/CD8^+^ T-cell ratio of group D (52 cases) was 0.150, showing no significance (*P* > 0.05).

## 4. Discussion

Lung cancer is a malignant tumor with high morbidity and mortality, among which 80%–85% of lung cancer patients have NSCLC. Despite the continuous progress of medical technology in recent years, the positive rate of NSCLC patients is still not high, and some studies have found that the 5-year SR is less than 20%. The immune system of the body, especially T cells, is closely related to the occurrence and development of tumors. Zylbermann et al. speculated that tumor-infiltrating lymphocytes can defend against tumors [36]. Tumor-infiltrating T cells are lymphocytes dominated by T cells that exist locally in tumors and can inhibit tumor growth, representing the body's anti-tumor immune response [37]. TILs with different densities have different prognostic effects on tumors. Studies have confirmed that certain types of CD3^+^ T and CD8^+^ T cells in TILs can inhibit tumor growth and have immune effects on tumor patients [38]. It was found that the total number of tumor-infiltrating T cells was related to the prognosis of the tumor due to the immune effect on the tumor, and the tumor-infiltrating T-cell subpopulation also had a strong relationship with the prognosis [39]. Tumor-infiltrating T cells are populations containing many different subsets of cells, which can be divided into different subsets according to different differentiated antigens, including 5 types, namely, CD3^+^, CD4^+^, CD8^+^, CD16^+^, and CD25^+^ [40]. Under normal circumstances, the ratios of TILs and T-cell subsets from different tumor sources are different, but the ratios remain constant and cooperate and restrict each other to maintain the normal immune function of the body. Higher or lower ratios of either of them will lead to immune disorders. Observing the status of tumor-infiltrating T cells in NSCLC patients can analyze their immune effect on tumors and analyze the development of tumors.

The results showed that 48 patients (26 males and 22 females) survived and 52 patients (33 males and 19 females) died at 3 years, with an positive rate of 48% (48/100). The 100 patients were divided into group S (*n* = 48) and group D (*n* = 52) according to 3-year survival. No great difference was indicated in age or sex between group S and group D, *P* > 0.05, which was comparable. HE staining of the pathological sections of 48 patients in group S showed lymphocyte infiltration, with a positive rate of 100%, including 0 cases of grade 0, 5 cases of grade 1 (10.42%), 16 cases of grade 2 (33.33%), and 27 cases of grade 3 (56.25%). In group D (52 cases), 7 cases did not have lymphocytic infiltration, and 45 cases had lymphocytic infiltration, with a positive rate of 86.54%, including 4 cases of grade 0 (8.89%), 10 cases of grade 1 (22.22%), 25 cases of grade 2 (55.56%), and 6 cases of grade 3 (13.33%). The total number of T-cell infiltrates in group S was higher than that in group D (*P* < 0.05). This finding indicates that tumor-infiltrating T cells have a certain immune effect on NSCLC patients. The higher the total number of T-cell infiltrates, the slower the tumor development and the higher the survival rate of patients.

Immunohistochemical staining of pathological sections of all patients with CD8, CD4, and CD25 mAbs showed that the average positive rate of CD8^+^ T-cell infiltration in group S (48 cases) was 72.1%, and in group D (52 cases), it was 47.6%. The average positive rate of the CD8^+^ subgroup in group S was higher than that in group D (*P* < 0.05). The average positive rate of CD4^+^ T-cell infiltration in group S (48 cases) was 15.3%, and in group D (52 cases), it was 11.7%. The average positive rate of CD4^+^ subsets in group S was superior to that in group D, and the difference was not substantial (*P* > 0.05). The average positive rate of CD25^+^ T-cell infiltration in group S (48 cases) was 13.2% and that in group D (52 cases) was 8.9%. The average positive rate of the CD25^+^ subgroup in group S was slightly higher than that in group D (*P* > 0.05). This finding indicates that the postoperative survival rate of NSCLC patients is related to T-cell infiltration, especially CD8^+^ cell infiltration, which has an immunosuppressive effect on local tumors. CD4^+^ and CD25^+^ also have certain immune effects on tumors, but the inhibitory activity is not strong, which may be related to the TNM stage of the disease, and the activity gradually increases with the development of the disease. However, it was found that the number of CD4^+^ cells is closely related to tumor growth [41], which was not found in this study and may be related to TNM stage and the differentiation degree of patients.

The percentages of CD8^+^ and CD4^+^ cells were 87.4% in group S (48 cases) and 59.3% in group D (52 cases), respectively (*P* < 0.05). The ratio of the CD8^+^ to CD4^+^ T-cell-positive percentage in group S (48 cases) was 4.71 on average. In group D (52 cases), the average was 4.07, and group S was higher than group D, with no notable difference (*P* > 0.05). The results showed that the sum of the CD8^+^ and CD4^+^ percentages was related to the postoperative survival of patients, which may be related to the immune effect of CD8^+^; the higher the CD8^+^ CD4^+^ value is, the stronger the immune effect of the tumor, and the higher the SR. Some studies have shown that the CD8^+^/CD4^+^ ratio can evaluate the balance of the immune system and reflect regulatory ability, but there was no evident difference between the two groups of patients in this study, which may still need to be confirmed by many studies [42]. The average ratio of CD25^+^ to CD8^+^ T cells in group S (48 cases) was 0.183, and in group D (52 cases), it was 0.187, showing no statistical significance (*P* > 0.05). The average ratio of CD25^+^ to CD4^+^ T cells was 0.863 in group S (48 cases) and 0.761 in group D (52 cases) (*P* > 0.05). The average ratio of CD25^+^ to (CD8^+^ + CD4^+^) T cells in group S (48 cases) was 0.151 and that in group D (52 cases) was 0.150 (*P* > 0.05). The results showed that there was no obvious relationship between CD25^+^/CD8^+^, CD25^+^/CD4^+^, CD25^+^/(CD8^+^ CD4^+^) and the SR of NSCLC patients. Nevertheless, there are still some limitations in this study. First, the sample size was small and single, requiring larger samples to verify the results. Second, the follow-up period was 3 years, and patients were not followed up for 5 years or longer. All these factors will lead to errors in exploring the tumor immunity effect of tumor-infiltrating T cells on NSCLC.

## 5. Conclusion

In summary, the postoperative survival rate of NSCLC patients was related to T-cell infiltration, especially CD8^+^ cell infiltration, which had an immunosuppressive effect on local tumors. In addition, it was also related to the sum of the CD8^+^ and CD4^+^ percentages. The higher the CD8^+^ + CD4^+^ value, the stronger the immune effect on the tumor, and the higher the SR of patients. Therefore, tumor-infiltrating T cells can inhibit tumor growth, which is related to the postoperative SR of NSCLC patients. In conclusion, tumor-infiltrating T cells had an immunosuppressive effect on tumor cells, and the total number and subsets of tumor-infiltrating T cells had a great impact on the survival rate of patients.

## Figures and Tables

**Table 1:** Main reagents used in the experiment.

Reagent	Detection	Company
CD8 monoclonal antibody (McAb)	CD8^+^ T cell	BD, USA
CD4 McAb	CD4^+^ T cell	
CD25 McAb	CD25^+^ T cell	

**Table 2:** Grade indicators of lymphocyte infiltration.

Level	Indicator
0	No lymphocyte
1	A small amount, approximately 1/3 of the field of vision
2	Distributed in clusters, accounting for approximately 1/3 to 2/3 of the field of vision
3	A lot, more than 2/3 of the field of view

## Data Availability

The data set used in this paper can be obtained from the corresponding author upon request.
